# The altered expression of neurofilament in mouse models and patients with spinal muscular atrophy

**DOI:** 10.1002/acn3.51336

**Published:** 2021-03-08

**Authors:** Charlotte Spicer, Ching‐Hua Lu, Francesco Catapano, Mariacristina Scoto, Irina Zaharieva, Andrea Malaspina, Jennifer E. Morgan, Linda Greensmith, Francesco Muntoni, Haiyan Zhou

**Affiliations:** ^1^ Department of Neuromuscular Diseases UCL Queen Square Institute of Neurology University College London London United Kingdom; ^2^ Trauma and Neuroscience Centre Blizard Institute Barts and The School of Medicine and Dentistry Queen Mary University of London London United Kingdom; ^3^ Neurology School of Medicine China Medical University and Hospital Taichung Taiwan; ^4^ Dubowitz Neuromuscular Centre Great Ormond Street Institute of Child Health University College London 30 Guilford Street London United Kingdom; ^5^ NIHR Great Ormond Street Hospital Biomedical Research Centre London United Kingdom; ^6^ Genetics and Genomic Medicine Teaching and Research Department Great Ormond Street Institute of Child Health University College London 30 Guilford Street London United Kingdom

## Abstract

**Objectives:**

To investigate the levels of neurofilaments (NFs) in transgenic mice and patients with spinal muscular atrophy (SMA), and to evaluate their efficacy as a biomarker in SMA.

**Methods:**

The levels of *NF* mRNA transcripts were measured by quantitative real‐time PCR in spinal cord from SMA mice. Blood levels of NF heavy chain (NfH) from mice and patients were measured by an in‐house ELISA method. The response of NFs to therapeutic intervention was analysed in severe SMA mice treated with morpholino antisense oligonucleotides.

**Results:**

Significant changes in *NF* transcript and protein in spinal cord and protein levels in blood were detected in SMA mice with severe or mild phenotypes, at different time points. A decrease in blood levels of NfH after antisense oligonucleotide treatment was only transient in the mice, despite the persistent benefit on the disease phenotype. A drastic reduction of over 90% in blood levels of NfF was observed in both control and SMA mice during early postnatal development. In contrast, blood levels of NfH were found to be decreased in older SMA children with chronic disease progression.

**Interpretation:**

Our results show that blood NfH levels are informative in indicating disease onset and response to antisense oligonucleotides treatment in SMA mice, and indicate their potential as a peripheral marker reflecting the pathological status in central nervous system. In older patients with chronic SMA, however, the lower NfH levels may limit their application as biomarker, highlighting the need to continue to pursue additional biomarkers for this group of patients.

## Introduction

Spinal muscular atrophy (SMA) is an autosomal recessive neuromuscular disease caused by mutations in the *Survival of Motor Neuron 1* (*SMN1*) gene.^1^ It is one of the most common genetic diseases in childhood and the leading genetic cause of infantile mortality.^2^ SMA patients experience progressive loss of motor neurons in the ventral anterior horn of the spinal cord, skeletal muscle denervation and paralysis of trunk and limb muscles. The SMN protein is essential in small nuclear ribonucleoprotein (snRNP) assembly and functions as the core component of the pre‐mRNA splicing machinery.^3^ Deficiency of this protein has been linked to morphological abnormalities in motor neuron bodies (somas) and axons in motor neurons cultured from SMA mice,^4^ and disrupted axonal extension and pathfinding in zebrafish.^5^ The major pathological hallmark of SMA in mouse models is the degeneration of motor neurons in selective segments of the spinal cord, following defects in neuromuscular junctions.^6^

Neurofilament proteins (NFs) are neuron‐specific cytoskeletal components belonging to the type IV intermediate filament family. They are assembled into a heteropolymer structure composed of three subunits of varying molecular mass: neurofilament light chain (NfL, 68 kDa), neurofilament medium chain (NfM, 150 kDa) and neurofilament heavy chain (NfH, 200 kDa).^7^ NFs play an important role in regulating the axonal diameter of myelinated axons, neuronal differentiation, axon outgrowth and regeneration.^8^, ^9^ NFs undergo various post‐translational modifications, in particular, phosphorylation which regulates their transport, function and degradation.^10^

Abnormal accumulations of NFs have been reported in several neurodegenerative diseases, including Amyotrophic Lateral Sclerosis (ALS), Charcot‐Marie‐Tooth, Parkinson’s Disease, Alzheimer Disease, giant axonal neuropathy, diabetic neuropathy and SMA.^11^ When axonal damage and neuronal death occur, NF proteins are released and delivered into cerebrospinal fluid (CSF), and detected in blood. Levels of NFs in CSF and blood have therefore been used as biomarkers for axonal injury, degeneration and neuronal loss in a number of neurodegenerative conditions, including SMA.^12^, ^13^, ^14^

Over the last few years, several studies have investigated the potential of NFs as biomarkers for SMA patients, with the rapid and successful clinical development of nusinersen, the first approved antisense oligonucleotide drug for SMA.^15^, ^16^ Grossly elevated plasma phosphorylated neurofilament heavy chain (pNF‐H) levels have been identified in infants with SMA type 1 compared to age‐matched controls.^13^ The raised pNF‐H levels were found to be inversely correlated with the age at the commencement of nusinersen and the severity of the clinical phenotypes. Moreover, the elevated pNF‐H levels were dramatically reduced after nusinersen treatment, indicating plasma pNF‐H levels are a promising biomarker for disease activity and response to nusinersen treatment.^13^ However, the same study also demonstrated that pNF‐H declined with advancing age in both untreated patients and in control children without SMA, raising questions on the role of pNF‐H as a biomarker in older patients. Moreover, other studies performed in SMA patients with milder clinical subtypes (type 2 and 3) did not find any difference in the levels of NFs in either children or adult patients compared to age‐matched controls.^17^, ^18^, ^19^ Finally, in older SMA patients NF levels in CSF and plasma show little or no response to nusinersen treatment,^17^, ^18^, ^19^ in striking contrast to the findings in the SMA type 1 infants. It is therefore important to expand our knowledge about the levels of NFs across different age ranges and determine how sensitive their measure could be in predicting the response of motor neurons to any effective treatment.

In this study, we used an in‐house ELISA method, developed and validated previously in ALS mice and patients,^20^, ^21^, ^22^ to quantify hyperphosphorylated (NfH^SMI34^) and variably‐phosphorylated (NfH^SMI35^) NfH levels in serum from SMA mice and from individuals affected by SMA type 2 and 3. To assess the correlation in expression of NFs in the CNS and in peripheral blood, NF mRNA transcripts and proteins were also measured in spinal cord samples from SMA mice, at different time points during early postnatal development. We included both the severe SMA type I‐like (SMA‐I) and the mild SMA type III‐like (SMA‐III) mice in this study, to understand the correlation between NF levels and SMA severity. Furthermore, in order to assess the potential of serum NFs as a CNS‐related outcome measure in response to efficacious treatment, we examined the effect of antisense oligonucleotide treatment on NF levels in SMA‐I mice.

## Materials and Methods

### SMA patients and healthy controls

The study was approved by the Berkshire Research Ethics Committee (REC reference 05/MRE12/32). Serum samples were supplied by the MRC Centre for Neuromuscular Diseases Biobank London (REC reference number 06/Q0406/33). Blood samples were collected from individuals following written informed consent from parents or legal guardians, from the SMA clinic at Great Ormond Street Hospital, London. All patients have a clinical diagnosis of SMA type 2 or 3 and a confirmed genetic diagnosis with homozygous genomic deletion in the *SMN1* gene. The patients recruited into this study were treatment naïve at the time when blood samples were collected. Control samples were obtained from healthy age‐matched children following written informed consent from parents or carers. All participating subjects are anonymous in this study.

### SMA mice

SMA transgenic mice, FVB. Cg‐Tg(*SMN2*)_2_Hung *Smn1* ^tm1Hung^/J (TJL005058), were used in this study.^23^ All the procedures conducted in mice were carried out in the Biological Services Unit, University College London Great Ormond Street Institute of Child Health, in accordance with the Animals (Scientific Procedures) Act 1986. Protocols were covered by Home Office project license. Antisense solution was injected subcutaneously as described previously.^24^

### Antisense oligonucleotides

The morpholino antisense oligomer PMO25 was manufactured by Gene Tools (https://www.gene‐tools.com/) with the sequence described previously.^24^, ^25^ PMO25 was synthesised in powder and dissolved in sterile water at a concentration of 10 *µ*g/*µ*L and stored at room temperature.

### RNA extraction and analysis in SMA mouse tissue

Total RNA was extracted from snap‐frozen spinal cord tissue collected from mice, using the RNeasy Mini kit (Qiagen). 500 ng RNA sample was used for cDNA synthesis using the high capacity cDNA synthesis kit (Applied Biosystems). Quantitative real‐time PCR was performed with the SYBR Green qPCR kit (Eurogentec). Primers were designed to specifically amplify mouse *Nefl*, *Nefm* and *NefH* transcripts (Table 1). *Gapdh* was used as the reference gene. Quantitative real‐time PCR was performed using the StepOne Real Time PCR System (Applied Biosystems). Quantification was based on the concurrent standard curve method.

**Table 1:** Sequences of primers used in this study.

Primer	Sequence (5ʹ to 3ʹ)	PCR product size
*mNefl*	F: GCGCCATGCAGGACACA	69 bp
	R: ACCTGGCCATCTCGCTCTT	
*mNefm*	F: AGCTGCAGTCCAAGAGCATC	130 bp
	R: AACTGCTGGATGGTGTCCTG	
*mNefh*	F: CTCCCAAAAATTCCCTCCAT	124 bp
	R: TCACCCGGATCTCTTCTGTC	
*mGapdh*	F: AGAGTGGGAGTTGCTGTTGAAGTC	133 bp
	R: CCTGGAGAAACCTGCCAAGTATG	

### Western blotting

Mouse spinal cord was lysed in buffer containing 75 mmol/L Tris‐HCl (pH 6.8) and 0.25% sodium dodecyl sulphate (SDS) supplemented with protease inhibitor cocktail (Roche Diagnostics). Protein concentration was analysed with a bicinchoninic acid (BCA) kit (Thermo Fisher Scientific). Fifty micrograms of total protein were loaded into 4–12% NuPAGE Bis‐Tris precast gels (Life Technologies). NfH protein (200 kDa) was probed by a rabbit anti‐NfH antibody (NF200, 1:500, Sigma‐Aldrich). *β*‐Tubulin was used as a protein loading control and was detected with a mouse anti‐tubulin monoclonal antibody (1:3,000; Sigma‐Aldrich). Membranes were probed with IRDye 800CW‐conjugated goat anti‐mouse and 680RD‐conjugated goat anti‐rabbit secondary antibodies (1:15,000, Li‐Cor). Blots were developed with Odyssey Imaging System (Li‐Cor).

### NfH ELISA

An in‐house ELISA developed previously was used in this study.^20^, ^21^ Plates were pre‐coated with mouse monoclonal anti‐NfH antibodies (SMI‐34R; 1:5000, Covance, USA), and blocked with 150 *µ*L Barb_2_EDTA buffer containing 1% BSA. Serum samples were incubated with Barb_2_EDTA buffer containing 0.5 mol/L urea for 1 h at room temperature (RT), in order to break up NfH aggregates. This was followed by incubation with detector antibody (polyclonal rabbit anti‐NfH; 1:1000, Sigma) and HRP swine anti‐rabbit reporter antibody (1:1000; DAKO). The absorbance was read on an Omega plate reader (Software version 1.02; BMG Labtech). Measurements were excluded if they had a coefficient of variation value higher than the assay limit (10%).

### Statistical analysis

Statistical analysis was performed using GraphPad Prism 8 (San Diego California USA). For NF transcript levels and blood NfH expression, non‐parametric Mann‐Whitney test was used for analysis between two groups, and one‐way analysis of variance (ANOVA) and post hoc *t* test were used for multiple groups. The correlations between serum NF levels and clinical motor function in SMA patients were analysed using linear regression. Statistical significance was set at *p* < 0.05. All data are presented as mean ± standard error of mean (SEM).

## Results

### Neurofilament transcripts were dysregulated in spinal cord from SMA mice

The transgenic SMA mouse strain used in this study, also known as the Taiwanese SMA mouse, can produce two types of SMA mice: the severe SMA‐I mice that carry two copies of human *SMN2* gene and have an average lifespan of 10 days; and the mild SMA‐III mice that carry 4 copies of human *SMN2* gene which have a normal lifespan, and the only phenotype of tail and ears necrosis commencing at approximately 3 weeks old.^24^ In the litter producing SMA‐I mice, half of the litter carrying the heterozygous genotype do not develop a disease phenotype and were therefore used as unaffected littermate controls (het control).^24^

To measure the expression of different *Nef* transcripts in the CNS in early postnatal development, spinal cord samples were collected from SMA‐I, SMA‐III and het control mice at postnatal day 5 (D5, an early symptomatic stage in SMA‐I and pre‐symptomatic stage in SMA‐III mice), 10 (D10, late symptomatic stage in SMA‐I and pre‐symptomatic stage in SMA‐III mice) and 20 (D20, symptomatic stage in SMA‐III mice). Quantitative real‐time PCR was used to measure the transcripts of mouse *Nefl*, *Nefm* and *Nefh*, relative to *Gapdh*.

**Figure 1:**
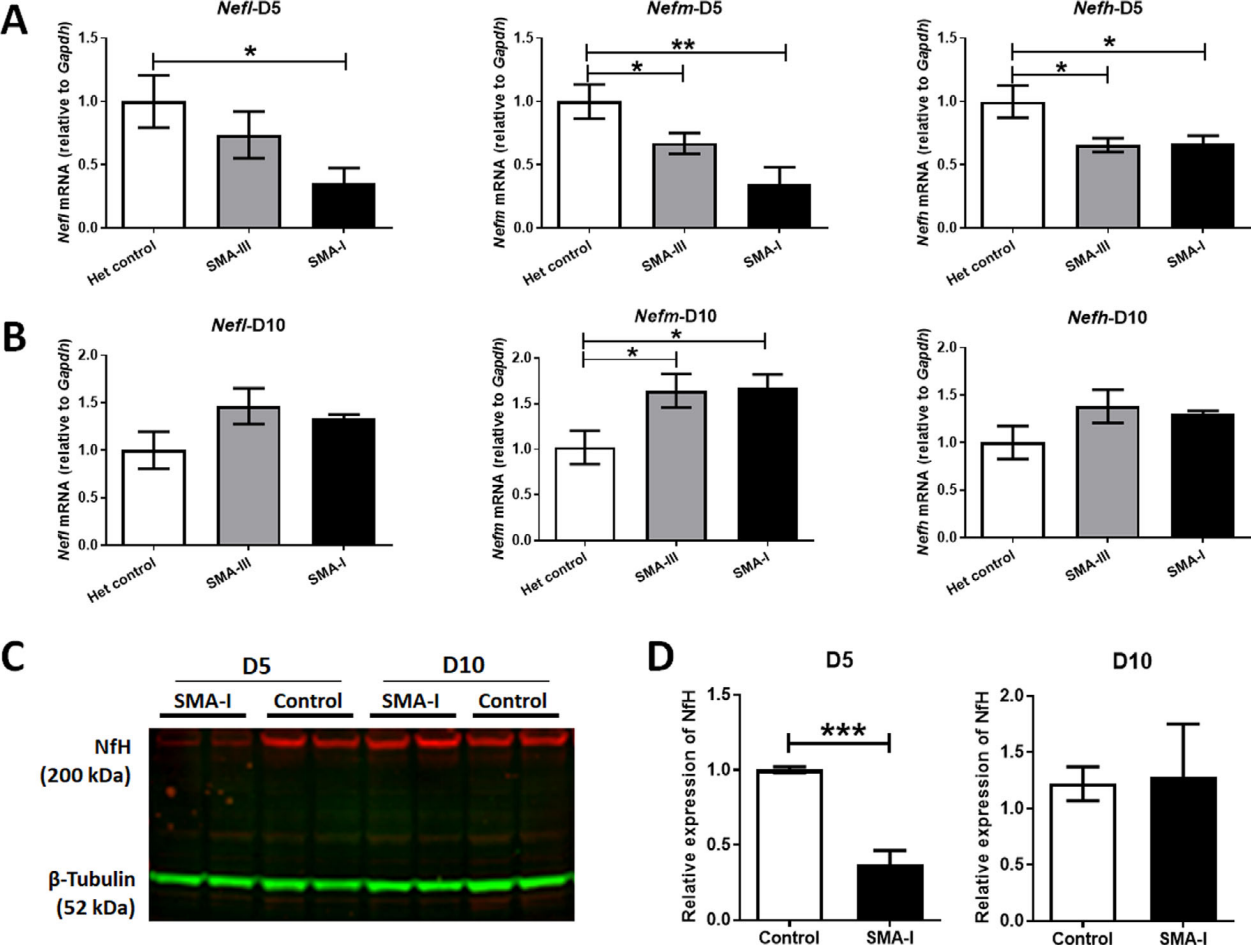
Expression of *Nef* mRNA transcripts in spinal cord tissues from SMA mice. Quantitative real‐time PCR analysis of *Nefh*, *Nefm* and *Nefl* transcripts, relative to *Gaphd*, were performed in spinal cord samples collected from the severe SMA type I mice (SMA‐I), mild type III mice (SMA‐III) and unaffected heterozygous littermate controls (Het control) at (A) postnatal day 5 (D5) and (B) postnatal day 10 (D10), respectively (*n* = 4/group). Data were analysed by one‐way ANOVA followed by post *t* test. (C) A representative image of western blotting of NfH (200 kDa) in spinal cord samples collected from SMA‐I and het control mice at postnatal day 5 (D5) and day 10 (D10). *β*‐Tubulin (52 kDa) was used as a loading control. (D) Semi‐quantification of the density of NfH protein bands, relative to *β*‐tubulin, in SMA‐I and control mice (*n* = 4/group). Data were analysed by unpaired *t* test. *p < 0.05, **p < 0.01, ***p < 0.001.

At day 5, a significant reduction in the expression of all the three *Nefs* was detected in the spinal cord of severe SMA‐I mice, with a 65% decrease in both *Nefl* (*p* < 0.05) and *Nefm* (*p* < 0.01) and a 33% reduction in *Nefh* (*p* < 0.05), compared to the het control group. Reductions in *Nefl*, *Nefm* and *Nefh* were also detected in SMA‐III mice, although to a lesser degree than in SMA‐I mice, with a decrease of 26%, 33% and 35%, respectively (Fig. 1A).

In contrast, at day 10, significant increases in *Nefm* transcripts in the spinal cord were detected in both SMA‐I (64%, *p* < 0.05) and SMA‐III mice (68%, *p* < 0.05). The levels of *Nefl* and *Nefm* transcripts were also increased although to a lesser extent in SMA‐I (34% for *Nefl* and 30% for *Nefh*) and SMA‐III mice (46% for *Nefl* and 38% for *Nefh*), compared to het control mice. These latter differences did not reach statistical significance (Fig. 1B).

### NfH protein was decreased in spinal cord from SMA‐I mice at the early symptomatic stage

To study NF expression at the protein level in spinal cord from SMA mice, we performed western blotting on NF heavy chain (NfH) in samples collected from SMA‐I and het control mice at postnatal days 5 and 10. At postnatal day 5, a significant 63% decrease (*p* < 0.0007) of NfH protein was detected in spinal cord from SMA‐I mice, compared to het control. However, there was no significant difference in NfH protein levels between SMA‐I mice and het controls at postnatal day 10 (Fig. 1C and D).

### Blood levels of NfH in SMA mice increased significantly at symptomatic stages

As high levels of phosphorylated NfH have previously been detected in neurodegenerative disorders,^11^ we measured hyperphosphorylated (NfH^SMI34^) and variably‐phosphorylated (NfH^SMI35^) NfH levels in serum from mice in each experimental group using a four‐layer sandwich ELISA. Blood levels of NfH proteins were examined at various time points, representative of different stages of the condition in SMA‐I and SMA‐III mice.

**Figure 2:**
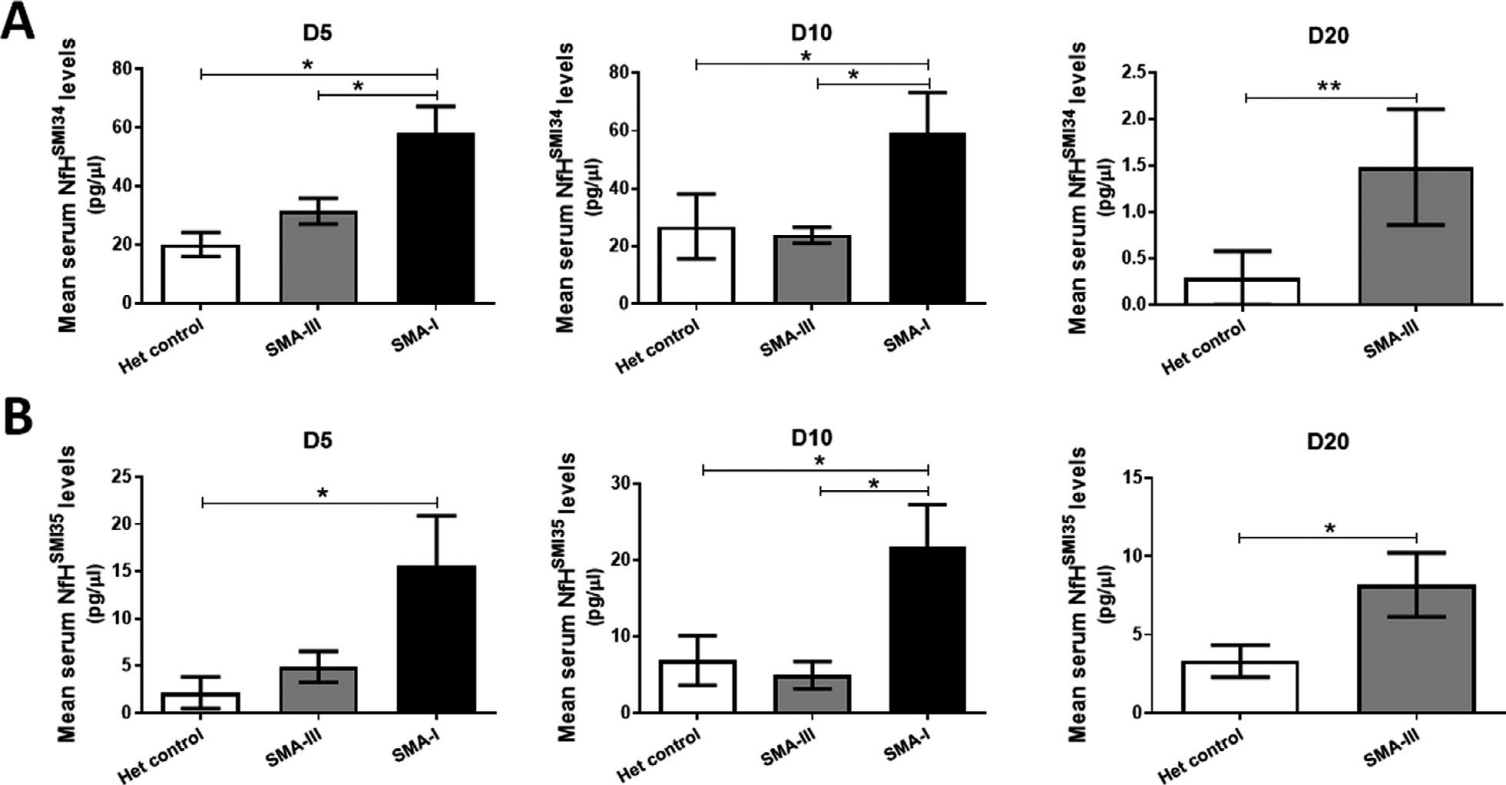
Quantification of blood NfH levels in control and SMA mice. In‐house ELISA analysis was used to quantify the levels of NfH^SMI34^ (A) and NfH^SMI35^ (B) in serum samples collected from SMA‐I, SMA‐III and het control mice at postnatal day 5 (D5), 10 (D10) and 20 (D20) (*n* = 4–6/group). Data were analysed by one‐way ANOVA followed by post *t* test. *p < 0.05, **p < 0.01.

At postnatal day 5, the serum levels (pg/*µ*L) of NfH^SMI34^ in SMA‐I mice (57.94 ± 9.24) were significantly increased compared to the levels in SMA‐III (31.40 ± 4.42, *p* < 0.05) and het control mice (20.06 ± 4.15, *p* < 0.05). This was also the case at postnatal day 10, with the levels in SMA‐I mice (59.35 ± 13.85) again significantly increased compared to the levels in SMA‐III (23.83 ± 2.78, *p* < 0.05) and het control (26.85 ± 11.17, *p* < 0.05) mice. The levels of NfH^SMI34^ were comparable between SMA‐III and het control at both day 5 and day 10, whilst a significant increase was detected at day 20 between these two groups (1.48 ± 0.62 vs. 0.28 ± 0.28, *p* < 0.01), the time point at which SMA‐III mice start presenting with a tail necrosis phenotype (Fig. 2A).

A similar pattern of change was also detected in the levels (pg/*µ*L) of the NfH^SMI35^ protein. Serum NfH^SMI35^ was significantly increased in SMA‐I mice compared to SMA‐III and het control mice at both day 5 (SMA‐I 15.53 ± 5.32, SMA‐III 4.90 ± 1.63, het control 2.14 ± 1.67, *p* < 0.05) and day 10 (SMA‐I 21.85 ± 5.38, SMA‐III 4.97 ± 1.79, het control 6.87 ± 3.22, *p* < 0.05). While no difference was detected between SMA‐III and het control at day 5 and 10, levels of serum NfH^SMI35^ were significantly increased in SMA‐III mice (8.18 ± 2.04) at day 20, compared to het controls (3.32 ± 1.03, *p* < 0.05) (Fig. 2B).

### AON treatment led to a short‐lived alteration of NF transcripts and protein levels in mice

To understand the response of NFs to antisense treatment in SMA, we treated SMA‐I mice with the previously reported 25‐mer morpholino antisense oligomer PMO25, which augments *SMN2* exon 7 splicing and restores the SMN protein by annealing to the ISS‐N1 element in the *SMN2* intron 7.^24^ A single dose of 40 *µ*g/g PMO25 via subcutaneous injection at postnatal day 0 induces a very robust response in SMA‐I mice and prolongs survival from 10 days to over 200 days.^24^ Spinal cord tissue and blood samples were collected from PMO25‐treated SMA‐I mice at postnatal day 5, 10 and 20.

**Figure 3:**
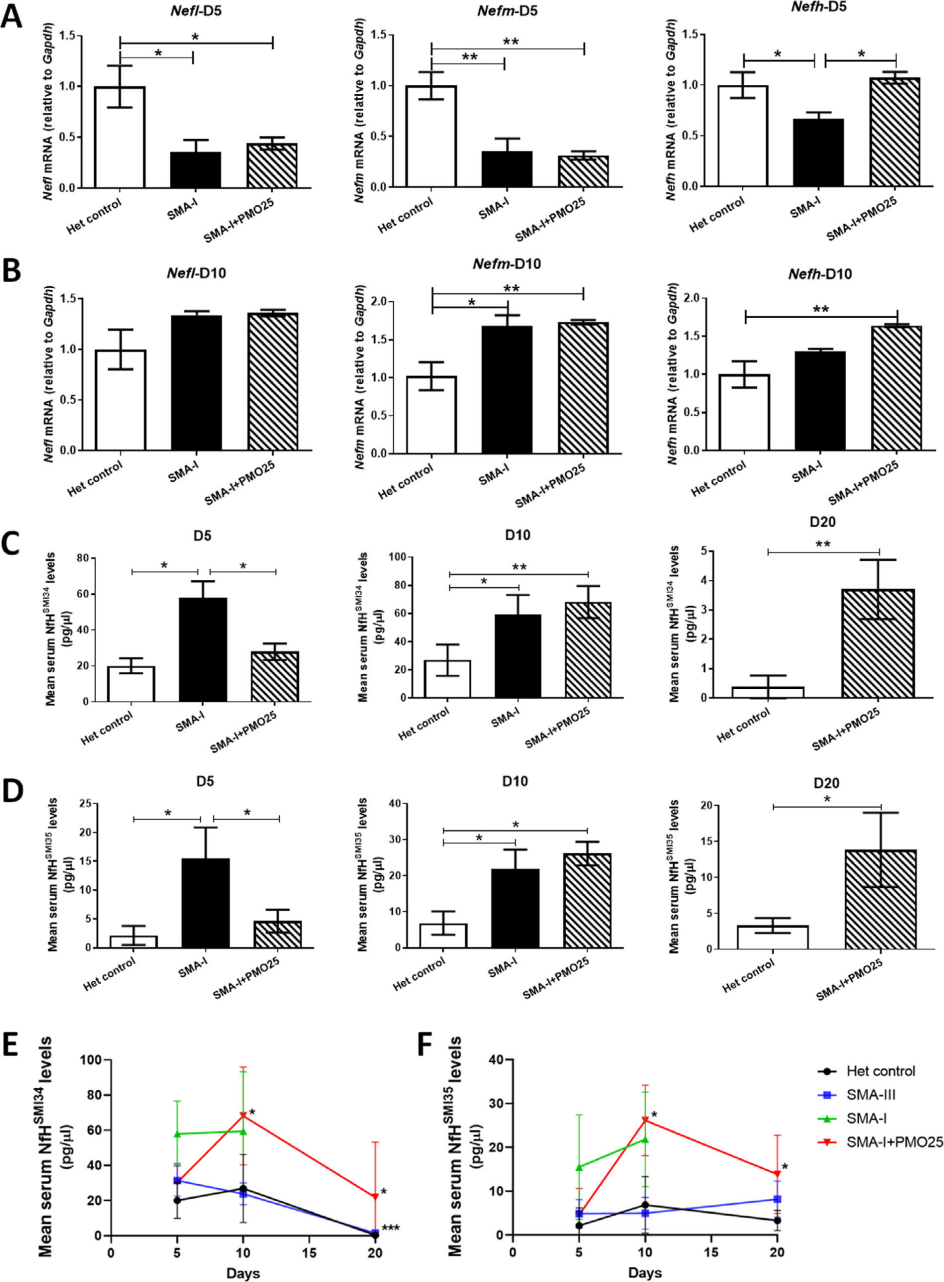
The response of *Nef* mRNA transcripts and NfH protein to antisense oligonucleotide treatment in the severe SMA mice. Quantitative real‐time PCR analysis of *Nefh*, *Nefm* and *Nefl* transcripts, relative to *Gaphd*, were performed in spinal cord samples collected from SMA‐I, SMA‐I treated with therapeutic antisense PMO25 (SMA‐I + PMO25) and het control at postnatal day 5 (A) and day 10 (B). ELISA analysis of serum levels of NfH^SMI34^ (C) and NfH^SMI35^ (D) in SMA‐I, SMA‐I + PMO25 and het control mice at day 5, 10 and 20. The dynamic changes of mouse serum NfH levels, (E) NfH^SMI34^ and (F) NfH^SMI35^, were detected in different experimental groups during early development at postnatal day 5, 10 and 20. Data were analysed by one‐way ANOVA followed by post *t* test, or unpaired *t* test (*n* = 4–6/group). Data were presented as mean ± SEM. *p < 0.05, **p < 0.01, ***p < 0.001.

The transcripts of *Nefl*, *Nefm* and *Nefh* mRNA in spinal cord at different time points were measured by qRT‐PCR. At day 5, while significant reductions were detected in all the three *Nefs* in SMA‐I compared to het control, only *Nefh* showed significant restoration to near normal level after PMO25 treatment (*p* < 0.05); we did not detect any response when analysing *Nefl* and *Nefm* (Fig. 3A). At day 10, increased mRNA transcripts were detected in *Nefl* (36%, *p* = 0.059), *Nefm* (73%, *p* = 0.004) and *Nefh* (64%, *p* = 0.005) in PMO25‐treated SMA‐I mice compared to the het control group. However, these increased levels of mRNA were similar to the levels in the spinal cords of untreated SMA‐I mice (Fig. 3B).

Serum NfH^SMI34^ and NfH^SMI35^ levels were also measured in blood samples collected from PMO25‐treated SMA‐I mice at different time points and compared to those in the untreated SMA‐I or het control groups. At day 5, both NfH^SMI34^ and NfH^SMI35^ levels (pg/*µ*L) showed a significant reduction in response to PMO25 treatment, by 52% (58 ± 9.2 in SMA‐I, 28 ± 4.5 in SMA‐I + PMO25, *p* = 0.001) in NfH^SMI34^ protein and 67% (15.5 ± 5.3 in SMA‐I, 4.6 ± 1.9 in SMA‐I + PMO25, *p* = 0.03) in NfH^SMI35^ protein (Fig. 3C and D). At day 10, however, serum NfH^SMI34^ and NfH^SMI35^ levels (pg/*µ*L) in PMO25 treated SMA mice (NfH^SMI34^ 68 ± 11, NfH^SMI35^ 26 ± 3.3) were similar to the untreated SMA‐I mice (NfH^SMI34^ 59 ± 13, NfH^SMI35^ 22 ± 5.4) and were significantly higher than those in the het control group (NfH^SMI34^ 26 ± 11, *p* = 0.02; NfH^SMI35^ 6.8 ± 3.2, *p* = 0.004). At day 20, significantly increased serum NfH^SMI34^ and NfH^SMI35^ levels were detected in PMO25‐treated SMA‐I mice (NfH^SMI34^ 3.7 ± 1.0, NfH^SMI35^ 13.8 ± 5.2) compared to het control mice (NfH^SMI34^ 0.38 ± 0.38, *p* = 0.019; NfH^SMI35^ 3.3 ± 1.0, *p* = 0.02) (Fig. 3C and D).

### Serum NfH levels changed dynamically during early postnatal development in control and SMA mice

To understand how NfH levels in blood vary with age during early postnatal development in both SMA and healthy controls, we compared the serum levels of NfH^SMI34^ and NfH^SMI35^ at postnatal day 5, 10 and 20, based on data obtained from the experiments conducted above.

In het control mice, mean serum NfH^SMI34^ levels stayed in the range of 20‐26 pg/*µ*L between days 5 and 10, then declined drastically by nearly 99% to 0.28 pg/*µ*L at day 20 (Fig. 3E).

In SMA‐III mice, the mean level of serum NfH^SMI34^ was 31 pg/*µ*L at day 5, slightly declined by 25% to 23 pg/μL at day 10, followed, similarly to control mice, by a drastic decline of approximately 94% to 1.48 pg/*µ*L at day 20 (Fig. 3E).

In SMA‐I mice, the mean level of serum NfH^SMI34^ was 58 pg/*µ*L at day 5, comparable to the levels at day 10. In PMO25‐treated SMA‐I mice, on the other hand, the mean level of NfH^SMI34^ was 32 pg/*µ*L at day 5, significantly increased by 2‐fold to 68 pg/*µ*L (*p* = 0.006) at day 10, then declined by 68% to 21.75 pg/*µ*L (*p* = 0.029) at day 20 (Fig. 3E).

In contrast to the drastic changes in serum NfH^SMI34^ in het control and SMA‐III mice, no significant changes were identified in serum NfH^SMI35^ in these mice. A slight fluctuation was observed between days 5 and 20 in the het control mice (mean levels ranging between 2.14 and 6.88 pg/*µ*L), and in SMA‐III mice (mean levels ranging between 4.90 and 8.18 pg/*µ*L), without any statistically significant difference (Fig. 3F). In SMA‐I mice, the mean levels of serum NfH^SMI35^ was slightly (but not significantly) increased from days 5 to 10. In PMO25‐treated SMA‐I mice, the change in mean levels of serum NfH^SMI35^ showed a similar trend as that of NfH^SMI34^. The mean level of NfH^SMI35^ was 4.6 pg/*µ*L at day 5, significantly increased by 5‐fold to 26 pg/*µ*L (*p* = 0.002) at day 10, then declined to 13.83 pg/*µ*L (*p* = 0.037) at day 20 (Fig. 3F).

### The levels of NfH in serum did not change in older SMA patients

To further evaluate the potential of serum NFs as noninvasive biomarkers for SMA patients affected by the chronic variants of the disease, we examined the levels of NfH^SMI34^ and NfH^SMI35^ in serum samples from a group of patients with SMA type 2 or 3 (*n* = 11), aged between 3 and 14 years old, and compared to healthy children (*n* = 8, age 4–8 years old). Summary of clinical information of the patients is presented in Table 2.

**Table 2:** Clinical data of the patients included in this study.

Sample ID	SMA type	Age at sample collection (years)	Gender	HFMS	SMN2 copy number
P1	3	14	M	34	NA
P2	3	7	F	39	NA
P7	3	8	M	39	NA
P8	3	8	F	36	NA
P9	2	4	M	19	NA
P3	2	9	M	4	2
P4	2	3	M	12	3
P5	2	12	M	21	3
P6	2	10	M	22	NA
P10	2	4	M	NA	NA
P11	2	7	M	NA	NA

Presented are the clinical subtype of SMA, age at sample collection, gender, *SMN2* copy number and Hammersmith Functional Motor Scale (HFMS) score of the patients.

NA, not available.

**Figure 4:**
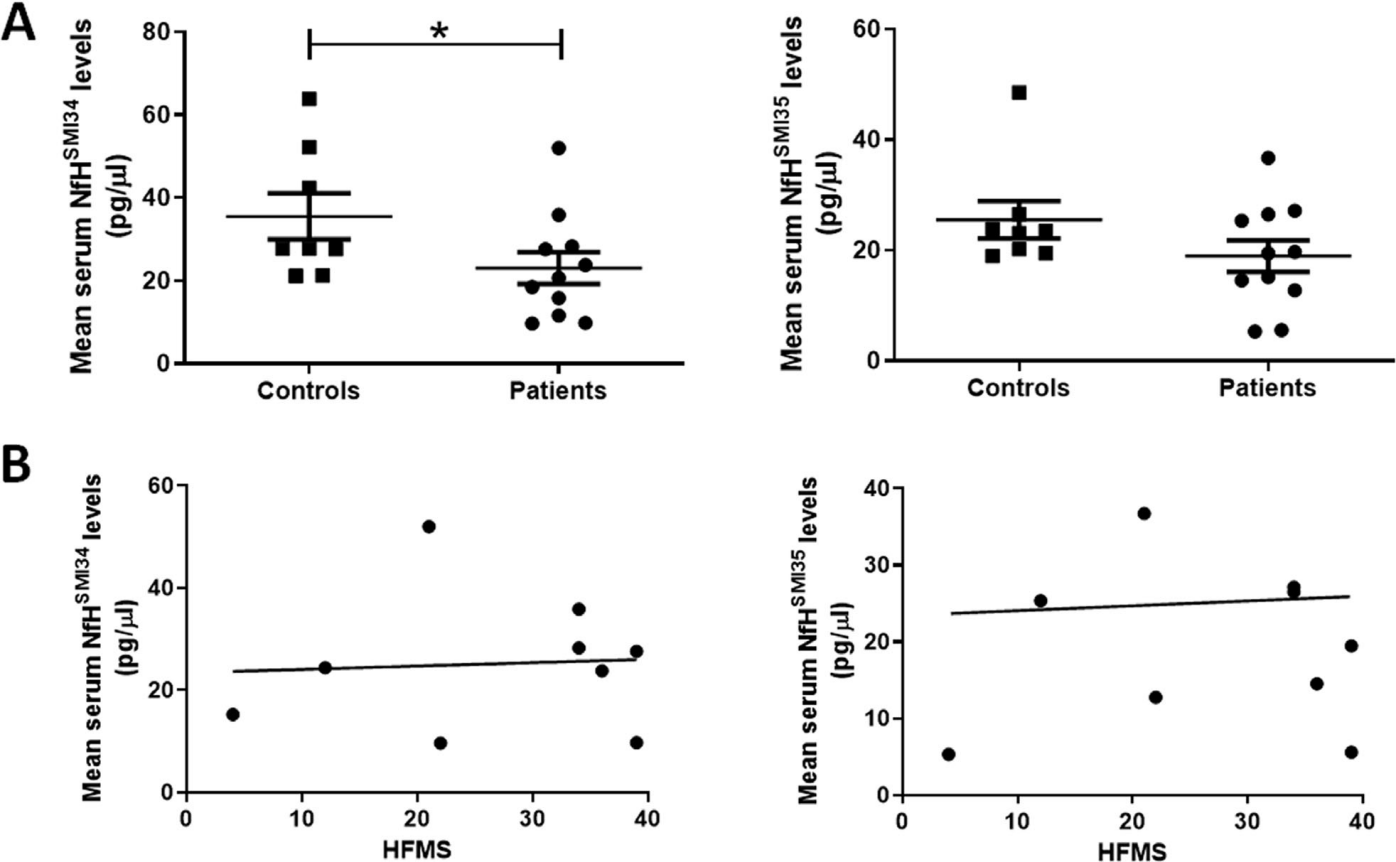
The serum levels of NfH in SMA patients and healthy controls. (A) Quantification of serum levels of NfH^SMI34^ and NfH^SMI35^was analysed by ELISA in SMA patients (*n* = 11) and age‐matched healthy controls (*n* = 8). Data were presented as mean ± SEM. *p < 0.05. (B) The correlations between the HFMS and levels of NfH^SMI34^ (*r* ^2^ = 0.028; *p* = 0.87) or NfH^SMI35^ (*r* ^2^ = 0.0039; *p* = 0.87) were analysed by linear regression. The regression line was presented. No significance was identified.

In this cross‐sectional study, we detected a slight but significant decrease in the levels (pg/*µ*L) of NfH^SMI34^ in SMA patients (23.06 ± 3.83) compared to healthy controls (35.52 ± 5.55, *p* = 0.0364). Serum levels of NfH^SMI35^ in SMA patients (18.95 ± 2.89) were also modestly reduced compared to controls (25.53 ± 3.42), although not significantly (Fig. 4A). We also correlated the serum levels of NfH to the motor functional ability based on the Hammersmith Functional Motor Scale (HFMS) total scores.^26^ The HFMS is an assessment of the physical abilities of SMA type 2 and type 3 patients with limited ambulation. The scale consists of twenty items with individual item scoring as 2 for unaided, 1 for performed with assistance and 0 for inability. The HFMS ranges from 0, if all the activities are failed, to 40, if all the activities are achieved.^26^ In this study, no correlation was identified between NfH^SMI34^ or NfH^SMI35^ serum levels and the HFMS scores (Fig. 4B).

## Discussion

In this study, we characterised the differential expression of neurofilaments in the spinal cord and blood of SMA mice with various disease severities, during early postnatal development. We also measured the response of these parameters to antisense oligonucleotide treatment in mice with the severe form of SMA, aiming to provide further evidence for the use of NF as a biomarker for SMA.

The degeneration of motor neurons is a hallmark feature of SMA. As motor neurons express high levels of NFs, it is not surprising that significant reductions in *Nef* mRNA transcripts of all three chains (*Nefl*, *Nefm* and *Nefh*) occurred in the spinal cord of severe type I SMA mice at the early symptomatic stage (postnatal day 5) (Fig. 1). However, to our surprise, there were no reduction in mRNA transcripts in end‐stage SMA‐I mice at day 10. Instead, mRNA transcripts were increased, by more than 50% for *Nefm* (Fig. 1B). This biphasic profile of NF expression in spinal cord at day 10 in SMA‐I was also confirmed at the protein level by western blotting (Fig. 1C). This suggests that there may be a compensatory mechanism involved in responding to the dramatic loss of NF in CNS, by increasing *Nef* transcripts in the spinal cord at the end stage of the severe SMA mice. It is acknowledged that western blot analysis was used to determine protein expression of only NfH in spinal cord from SMA mice in this study. This was to correlate with blood levels of NfH for biomarker evaluation. It would be useful to measure the expression of NfL or NfM proteins in spinal cord of SMA mice when validating the blood levels of these two proteins in future biomarker studies.

The change in *Nef* transcripts in response to antisense treatment appeared to be chain‐specific. In PMO25‐treated SMA‐I mice, at day 5, only the heavy chain transcript *Nefh* showed a sensitive response to antisense treatment, with levels restored to near normal levels (Fig. 3A). A recent study in severe SMA mice showed that the blood levels of NF‐L were maximally increased at postnatal day 1 and pNF‐H levels were increased at postnatal day 3.^14^ This may explain why the light chain transcript *Nefl* did not respond to antisense treatment as damages to the NF‐L expressing axons already occurred when mice were treated after birth, while the pNF‐H expressing axons were still intact at postnatal day 1 when mice were treated, and thus, responded sensitively to the treatment.

In the present study in SMA mice, there were increased blood levels of NfH^SMI34^ and NfH^SMI35^ in both the early (day 5) and late symptomatic (day 10) stages in the severe type I group. In the mild SMA‐III mice, no change in the blood levels of these proteins was detected at the pre‐symptomatic stage (day 5 or day 10). However, there were significantly increased levels of NfH^SMI34^ and NfH^SMI35^ at day 20, the early symptomatic stage in SMA‐III. These results suggest that blood levels of NfH^SMI34^ and NfH^SMI35^ are altered at the early symptomatic stages in both the severe SMA‐I and the mild SMA‐III mice, and may therefore be a useful biomarker in SMA‐I and ‐III mice at the early disease stage (Fig. 2). Our finding is further supported by a recent study where blood NF levels were found to be increased in the severe neonatal SMA‐I mice as early as postnatal days 1–3, associated with a rapid postnatal axon degeneration.^14^ Moreover, compared to mRNA and protein levels in spinal cord, the absolute levels of NfH in peripheral blood are more reliable and accessible as protein biomarker for SMA.

Blood levels of NfH^SMI34^ and NfH^SMI35^ responded sensitively to the efficacious antisense drug treatment in the severe SMA‐I mice (Fig. 3). However, this response was short‐lived, and the levels of NfH bounced back shortly after, even though the disease phenotypes, including survival and motor functions, continue to improve in the treated mice.^24^ Our previous studies have shown that a single‐dose of PMO25 treatment can markedly increase SMN protein in the brain and spinal cord and the maximum effect is sustained at both 10 and 20 days after the single‐dose injection.^24^ Therefore, it is unlikely that the transient decrease in NfH blood levels at 10 days after PMO25 treatment is related to the single‐dose nature of the intervention. While this may suggest an incomplete rescue with a single‐dose of PMO25 treatment in the severe SMA mice, it also implies that blood NfH levels are sensitive in reflecting the neuropathological progression in this model.

Dynamic levels of blood NF proteins were observed during early postnatal development in both the unaffected control and SMA mice in our study. In control mice, a slight fluctuation of NfH^SMI34^ levels was observed between day 5 and day 10, then the levels drastically declined by 99% at day 20 (Fig. 3E). A similar trend was also observed in the mild SMA‐III mice and PMO25‐treated SMA‐I mice, where blood levels of NfH^SMI34^ declined between day 10 and 20, by 94% and 68%, respectively (Fig. 3E). Moreover, our results showed that NfH^SMI34^, the hyperphosphorylated forms of NF, is more sensitive than NfH^SMI35^, the phosphorylated forms of NF, in reflecting the dynamic changes of blood NfH levels during the early stage development (Fig. 3E and F). This result further indicates a strong association of NfH phosphorylation with the dynamic axonal cytoskeletal network during the early development of nervous system.

In line with our data from the SMA mouse model, a similar observation has also been reported in both healthy children and those with SMA, where blood phosphorylated NF‐H (pNF‐H) levels appear to decline with age. The median pNF‐H level in symptomatic infants with SMA (<1 year, 15,400 pg/mL) is approximately 10 times of that in age‐matched non‐neurological disease controls (1510 pg/mL). In children without SMA, the level declined over 90% from infants aged <1 year to those aged 1–18 years (124.5 pg/mL).^13^ Blood pNF‐H concentration also declined over time in nusinersen and sham control‐treated SMA patients, although in the nusinersen treated group the pNF‐H levels declined more rapidly while in the sham control group the levels showed a gradual decline over the study duration.^13^ Similarly, in our study NfH protein levels displayed a dynamic expression over time and a drastic decrease during early postnatal development, in both control and SMA mice. Our study provides further information on the profile of NF changes over time that needs to be taken into account to distinguish this from the response to drug treatment.

It has been previously reported that blood pNF‐H levels in older patients (between 1 and 18 years old) are comparable to healthy individuals.^13^ In a separate study performed in SMA type 3 patients, baseline CSF neurofilament levels (pNF‐H and NFL) were in the same range as control.^17^ There was no significant correlation between the change in motor functions and that of neurofilaments over time.^17^ In another study performed in adult SMA patients, no significant changes in levels of pNF‐H were detected in CSF or blood either at baseline or during loading with nusinersen.^19^ In line with previous reports, the results of our study on blood NfH protein levels at baseline in 11 paediatric patients with SMA type 2 and 3 showed no increase in NfH protein levels in blood. Instead, a slight but significant reduction of NfH^SMI34^ was identified in these chronic SMA patients compared to age‐matched healthy controls, although the change was not associated with clinical motor function as assessed by the HFMS (Fig. 4). This finding possibly reflects the exhaustion of motor neuron pools in the CNS during chronic disease progression and provides further evidence for the limitation of the NfH protein as a biomarker for disease progression in older SMA patients.^27^ The finding also echoes previous observations in ALS patients with a fast‐progressing rate, whose blood NfH decreased over 15 months after recruitment to the study.^22^

Taken together, the results of this study provide further evidence of the role that blood NfH can play as a biomarker of disease onset, disease progression and onset response to drug treatment in SMA mice. Our cross‐sectional data in older SMA children indicate differences from the striking elevation observed in young severe type 1 SMA patients, and this might limit its usage as a biomarker for this group of patients, although more longitudinal data and control values from healthy children of different ages are still needed. Our study, therefore, highlights the need to identify additional biomarkers for older SMA patients with chronic disease progression.

## Conflicts of Interest

F.M. has served on scientific advisory boards for Sarepta, Pfizer, Roche, Biogen, and Avexis, receives research support from Biogen, and has received funding for trials from Sarepta, Avexis, Biogen, PTC, Wave, Roche, and Sarepta Therapeutics. M.S. has served on scientific advisory boards for Roche, Biogen and Avexis, and has received funding for trials from Roche and Biogen. No conflicts of interest from the other authors.
